# Effects of Different Vitrification Solutions and Protocol on Follicular Ultrastructure and Revascularization of Autografted Mouse Ovarian Tissue

**DOI:** 10.22074/cellj.2021.6877

**Published:** 2020-04-22

**Authors:** Mohammad Mahmoudi Asl, Reza Rahbarghazi, Rahim Beheshti, Alireza Alihemmati, Mohammad Reza Aliparasti, Ali Abedelahi

**Affiliations:** 1. Stem Cell Research Center, Tabriz University of Medical Sciences, Tabriz, Iran; 2. Department of Veterinary, Shabestar Branch, Islamic Azad University, Shabestar, Iran; 3. Department of Anatomical Sciences, Tabriz University of Medical Sciences, Tabriz, Iran; 4. Department of Immunology, Tabriz University of Medical Sciences, Tabriz, Iran; 5. Department of Reproductive Biology, Faculty of Advanced Medical Sciences, Tabriz University of Medical Sciences, Tabriz, Iran

**Keywords:** Angiogenesis, Cryopreservation, Graft, Mouse, Ovary

## Abstract

**Objective:**

Many attempts have been made to preserve fertility by improving the cryopreservation of the ovarian tissue. This current studyaimed to improve of direct cover vitrification (DCV) protocol on follicular preservation and angiogenesis in autografted ovarian tissue.

**Materials and Methods:**

In this experimental study, sixty five female Balb/c mice (5-6 week-old) were anesthetized and their ovaries were dissected. The left ovaries were vitrified by DCV solution, thawed by descending concentrations of sucrose, and then autografted subcutaneously. The right ovaries were autografted with no vitrification procedure prior to transplantation. The animals were sacrificed under anesthesia on the 7thday after transplantation to obtain ovarian tissue. Follicular quality was assessed by histological and ultrastructure observations, and angiogenesis was examined by immunohistochemical staining and real-time polymerase chain reaction (PCR) analysis.

**Results:**

The histological and ultrastructure features of the follicles preserved well after vitrification of the ovarian tissue by 10% ethylene glycol (EG) and 10% dimethyl sulfoxide (DMSO). Revascularizationwas manifested prominently in the DCV1-vitrified/grafted ovaries by von Willebrand factor (*vWF*) and alpha smooth muscle actin (*α-SMA*) immunostaining. The ovarian tissue vitrified in DCV1 protocol had higher expression levels of angiopoietin-2 (Ang-2) and vascular endothelial growth factor (*VEGF*) 7 days after autotransplantation (P<0.01).

**Conclusion:**

These findings suggest that DCV with 10% of both EG and DMSO, is an effective cryopreservation solution for preservation of good quality follicles as well an upregulation of angiogenic factors after ovarian tissue transplantation.

## Introduction

Ovarian tissue cryopreservation and grafting are appropriate strategies for fertility preservation in women undergoing radiotherapy and chemotherapy (1). The cryopreservation process and cryoprotectant (CP) toxicity are inevitable aspects of ovarian tissue cryopreservation (2). Vitrification is a simple and efficient procedurefor preservation of the follicle quality in cryopreservation of ovarian tissue (3, 4). However, vitrification requires high concentrations of CPs and fast cooling rates, which can cause cell injury and follicle loss (5). Both ethylene glycol (EG) and dimethyl sulfoxide (DMSO) as CP agents have been used commonly in cryopreservation of the ovarian tissue, because of their low toxicity and rapid permeability (6, 7). Youm et al. (7) proposed that combining EG and DMSO through a stepwise increase in concentration could improve follicle preservation quality due to its lowertoxicity. Many attempts have been made to optimize vitrification protocols through assessing appropriate cryopreservation techniques, CP agents and concentrations, cryopreservation devices, equilibration, and warming times (7-10). Recently, many studies have focused on improving cryopreservation protocols by accelerating the cooling rate using ultra-rapid vitrification, such as DCV (10, 11). Our previous study illustrated that DCV is an efficient method for preserving ovarian tissue (12, 13).Transplantation of ovarian tissue is an excellent experimental model for evaluating the effects of CP agents on follicular viability and development.

A subcutaneous grafting site was selected based on Shubert’s results (14). He reported that ovarian tissue was transplanted subcutaneously for accessible monitoring of angiogenesis and follicular morphology, which allowed for the preservation of ovarian integrity. The most important process in the early stages of ovarian transplantation is revascularization, which is regulated by angiogenic factors (15). It has been observed, however, that transplantation of ovarian tissue is subject to hypoxia that leads to follicle loss (16). Therefore, detection of angiogenic factors in vitrified ovaries is crucial for monitoring and evaluating follicular development after transplantation.

Several ligands and receptors participate actively in angiogenesis signaling pathways including VEGF, Ang and tyrosine kinase (17, 18). Angiogenesis is primarily controlled by VEGF, which is produced mainly by theca and/or granulosa cells in ovarian tissue (19-21). VEGF is an endothelial cell mitogen that regulates neovascularization and vascular permeability in grafted ovarian tissue (19), and has been demonstrated to regulate follicular growth as well as folliculogenesis (20, 21). Ang-2 is another angiogenesis factor that destabilizes endothelial-endothelial cell connections, resulting in the migration of endothelial cells and promoting further angiogenesis (22).

In addition, vWF is a glycoprotein expressed exclusively in endothelial cells, and α-SMA is a marker of mature pericyte cells, which stabilize new blood vessels and are considered as early signs of neo-angiogenesis within vitrified/grafted ovaries (18, 23).

There are still many obstacles and limitations in DCV for preserving fertility in patients undergoing chemoor radiotherapy for cancer. In this regard, the aim of this study was to evaluate the influence of different CP agents on autografted mouse ovarian tissue viability and revascularization using follicular morphology and ultrastructure, expression of angiogenesis factors, and endothelial and pericytes cell markers.

## Materials and Methods

### Chemicals

All chemicals in this experimental study were purchased from Sigma-Aldrich (Sigma Chemical Co., Deisenhofen, Germany) unless otherwise mentioned.

### Animals and ovarian tissue preparation

Sixty-five female Balb/c mice (5-6 week- old) were purchased from Tabriz University of Medical Sciencesand housed in standard conditions (12-hours light/12-hours dark, 22-25˚C and 55% humidity), according to the International Animal Care and Use Committee (IACUC) instructions, and were given free access to food and water. The animals were anesthetized with an intraperitoneal injection of ketamine (80 mg/kg IP) and xylazine (10 mg/kg IP).Then the ovaries were dissected through a small dorsolateral incision. The left ovaries were vitrified while the right ovaries were not vitrified as controls. The vitrified/thawed ovaries or non-vitrified ovaries were autografted into subcutaneous pockets in the lateral flank and placed on the lumbar muscles.

### Direct cover vitrification procedure

Our direct cover vitrification (DCV) protocol was a modified version of the one used by Zhou et al. (11). The CP and warming solutions were prepared in Dulbecco’s phosphate-buffered saline (DPBS).Three concentrations of the CP solution were prepared as follows:

i. 5% EG+5% DMSO+0.5 M sucrose+20% FBS (CP1) ii. 10% EG+10% DMSO+0.5 M sucrose+20% FBS (CP2) iii. 15% EG+15% DMSO+0.5 M sucrose+20% FBS (CP3)

The vitrified samples were prepared as follows:

i. Ovaries were vitrified sequentially to the CP1 and CP2 solutions for 12 minutes at room temperature (DCV1). ii. Ovaries vitrified sequentiallyto the CP1 and CP3 solutions for 12 minutes at room temperature (DCV2). iii. Ovaries equilibrated vitrified sequentiallyto the CP2 and CP3 solutions for 12 minutes at room temperature (DCV3).

iv. Ovaries vitrified sequentially to the CP1, CP2 and CP3 solutions for 12 minutes at room temperature (DCV4)

The surrounding vitrification medium was removed, and then, ovarian tissue was placed in a 1.8 plastic standard cryovial with a minimum volume of the vitrification solution and liquid nitrogen was applied directly in the cryovial (DCV). The cryovials were placed into a tank of liquid nitrogen (196 ˚C) and kept for one week.

### Thawing process

The cryovials containing vitrified ovaries were thawed in nitrogen vapor for 30 seconds, at room temperature for 30 seconds and then were put into a 38˚C water bath for 60 seconds. The ovarian tissue was suspended in 1 ml descending concentrations of sucrose (1, 0.5 and 0.25 M) and DPBS for 10 minutes. To evaluate the vitrification toxicity, a group of control ovaries were exposed to all stages of vitrification and thawing procedures except for being plunged into liquid nitrogen.

### Histological evaluation

The ovaries (n=5 from each groups) were fixed in 10% formalin-buffered solution, dehydrated in serial alcohol washes, clarified with xylene, embedded in paraffin wax, and sequentially sectioned at 5 μm thickness. The 10^th^ section of each ovary was mounted on glass slides (five sections from each sample), stained with hematoxylineosin (H&B) solution and observed under a light microscope at a magnification of ×400 in 10 fields for each sample. The ovarian follicles with visible nuclei in the oocyte were noted at various stages of development. Primordial follicles were characterized by a single layer of flattened granulosa cells surrounding the oocyte; primary follicles had a single layer of cuboidal granulosa cells around the oocyte; preantral follicles were characterized by two or more layers of cuboidal granulosa cells and no antrum; and antral follicles were characterized by the presence of an antrum filled with follicular fluid. Follicular quality was assessed as normal, having intact oocyte and regular granulosa cells, or degenerated, with cytoplasmic vacuolization, detachment of oocyte and granulosa cells and irregular granulosa cells with pyknotic nucleus (24).

### Ultrastructureevaluation

Both fresh and vitrified ovaries (n=3 from each groups) were fixed in 2.5% glutaraldehyde (TAAB Laboratories Ltd., Berkshire, UK) in phosphate-buffered saline (PBS, pH=7.4) for 2 hours at room temperature, washed in PBS, then post-fixed in 1% osmium tetroxide (TAAB Laboratories Ltd., Berkshire, UK) in the same buffer for 2 hours at 4˚C. After washing in PBS, the samples were dehydrated in ascending concentrations of ethanol, placed in propylene oxide, and embedded in Epon 812 (TAAB Laboratories Ltd., Berkshire, UK). The samples were sectioned and 0.5 μm sections (semi-thin sections) were stained with toluidine blue and observed under light microscope. Thin sections (70 nm) were prepared using glass blades and placed on copper grids, stained with uranyl acetate and lead citrate (TAAB Laboratories Ltd., Berkshire, UK), and evaluated by transmission electron microscope (Zeiss, Germany). The granulosa cells and oocytes were evaluated by the integrity of the cytoplasmic and nuclear membranes, the number and size of the vesicles and the structure of the cytoplasmic organelles.

### Immunohistochemical detection of endothelial and pericyte cells

The endothelial and pericyte cells were stained to identify new blood vessels in vitrified/grafted ovaries (n=3 from each group). The 5μm paraffin sections (three serial sections from each sample) were deparaffinized with xyloland rehydrated in graded alcohol series (Merck, Germany). The sections were incubated with hydrogen peroxide (3%) inmethanol for 30 minutes at room temperature to block endogenous peroxidase. After autoclaving in the citrate buffer, the slides were incubated with the primary antibodies for 30 minutes:1/100 antivWF (Dako, Denmark) for staining endothelial cells, and 1/100 anti-α-SMA (Dako, Denmark) for staining smooth muscle cells. The slides were washed with PBS, and stained with the EnVision+Dual Link System HRP kit (Dako, Denmark), 3-Diaminobenzidine (DAB).

Each specimen was observed under a light microscope (×400) (Nikon, Japan). Single or clusters of endothelial cells positive for yWF (brown dye), were considered indicative of vessels formation. In the current experiment, the results from treatment groups were compared with those of the intact ovarian tissue from the control mice. All immunohistochemical analyses were done in three independent experiments.

#### Real-time polymerase chain reaction procedure

As angiogenesis-related genes were expected to be expressed following successful transplantation of ovarian tissue in the mice, VEGF and Ang-2 primers were designed for evaluation of the genes. For this purpose, all fresh and vitrified/grafted ovaries were immediately frozen in liquid nitrogen and stored at -196˚C for real-time polymerase chain reaction (PCR) analysis. The ovaries were collected for RNA extraction by Trizol Reagent (Invitrogen, USA) according to the manufacturer’s recommendations. The specimens were treated with RNase-free DNase and single-stranded cDNAs and were synthesized by incubating 1 μg of isolated RNA. The realtime PCR analysis was carried out by the Corbett Life Science (Rotor-Gene 6000) System and Fast Start SYBR Green Master (Roche). Primer sequences for VEGF and Ang-2 are outlined in Table 1.

**Table 1:** Primers used for the real-time polymerase chain reaction assay


Gene name	Primer sequence (5ˊ-3ˊ)	Length (bp)

VEGF-a	F: GACAGAAGGAGAGCAGAAGTCC	223
	R: CATGGTGATGTTGCTCTCTGAC	
Ang-2	F: TGACGAGCTGGAGAAGAAGC	236
	R: TGGAGTTGGGGAAGGTCAGT	
β-microglobulin	F: CCTGGTCTTTCTGGTGCTTG	171
	R: CCGTTCTTCAGCATTTGGAT	


Real-time PCR amplifications were performed using the following program: denaturation of cDNA (1 cycle at 95˚C for 10 minutes), amplification (40 cycles at 95˚C for 15 seconds, 57˚C for 30 seconds and 63˚C for 38 seconds), and melting curve analysis (1 cycle at 60 to 95˚C with 1˚C/seconds). The mRNA expression levels were normalized by β-microglobulin (β-mg) and the quantification was evaluated using the 2^(∆∆Ct)^ method. The assay was performed in triplicate.

#### Statistical analysis

Data was analyzed using SPSS 24 (IBM, International Business Machines Corp., New Orchard Road Armonk, New York). Quantitative data is reported as means ± SD. The normality of data was evaluated using theKolmogrov- Smirnov test, and the homogeneity of variance was assessed using Levene’s test. Differences between the groups were evaluated using one-way analysis of variance (ANOVA). The results of real-time PCR were analyzed using the independent-samples t test as well as the Wilcoxon test. A value of P<0.05 was considered statistically significant.

#### Ethical consideration

All applicable international, national and institutional guidelines for the care and use of animals were followed by the IACUC of Tabriz University of Medical Sciences (No. 2004-0405). This article does not contain any studies with human participants performed by any of the authors.

## Results

### Histological examination

**Fig.1:**
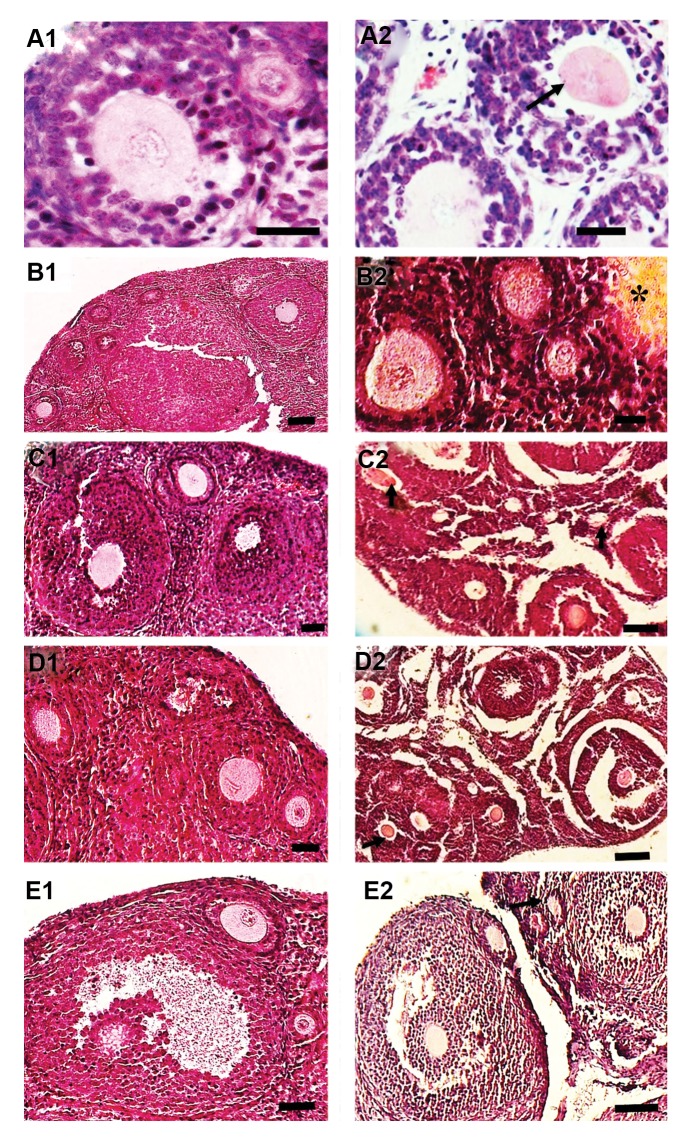
Histological images of mouse ovarian tissue. **A1.** Intact, **A2.** Fresh grafted, **B1.** Vitrified with DCV1, **B2.** Vitrified with DCV1/grafted, **C1**. Vitrified with DCV2, **C2.** Vitrified with DCV2/grafted, **D1.** Vitrified with DCV3, **D2.** Vitrified with DCV3/grafted, **E1.** Vitrified with DCV4, and **E2.** Vitrified with DCV4/ grafted. The follicular integrity and stromal tissue structure was well-preserved in the fresh and vitrified ovaries before transplantation. In the ovarian tissues vitrified with DCV2, DCV3 and DCV4 after transplantationmore signs of cryodamage, such as cytoplasmic retraction, shrinkage of the oocyte (black arrow) and fibrotic tissues (star), were observed (scale bar: 50 μm). DCV; Direct cover vitrification.

The morphology of primordial and primary follicles from vitrified ovarian tissue was well preserved, but some cryoinjury such as detachment of oocytes and granulosa cells was observed in the vitrified/grafted groups (Fig.1). After transplantation, the morphology of preantral and antral follicles was preserved significantly better in the ovaries from vitrified/grafted with DCV1 in comparison with the other vitrified/grafted groups (Fig.1B). Preantral and antral follicles from ovarian tissue vitrified with DCV2, DCV3, and DCV4 after transplantation showed numerous ultrastructural alterations, such as oocyte shrinkage, numerous cytoplasmic vacuoles, stromal fibrosis, and detachment of the oocyte from granulosa cells (Fig.1C-E).

### Ultrastructure analysis

**Fig.2:**
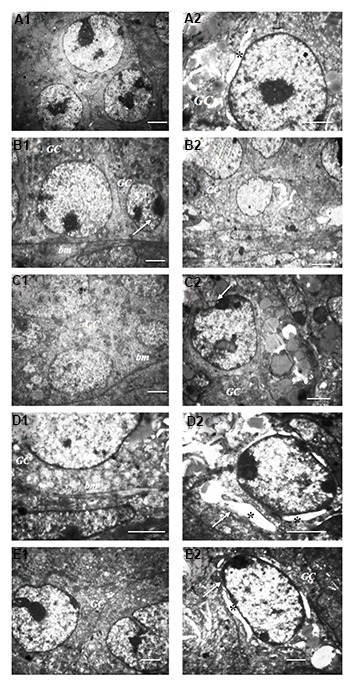
Ultrastructure of the antral follicles in murine ovarian tissue. **A1.** Intact, **A2.** Fresh grafted, **B1.** Vitrified with direct cover vitrification1 (DCV1), **B2.** Vitrified with DCV1/grafted, **C1.** Vitrified with DCV2, **C2.** Vitrified with DCV2/grafted, **D1.** Vitrified with DCV3, **D2.** Vitrified with DCV3/grafted, **E1.** Vitrified with DCV4, and **E2.** Vitrified with DCV4/grafted. The ultrastructures of cytoplasmic organelles in vitrified ovarieswere well-organized before transplantation. Granulosa cells (GC) hadnormal basement membrane (bm) and normal nucleus (N). After transplantation,more signs of damage were observed in vitrified ovaries including nucleus shrinkage in granulosa cells (white arrow) and perinuclear space (PS) (star) (scale bar: 500 μm).

The ultrastructure of the follicles in vitrified ovaries showed a well-developed cytoplasmic organelle, round mitochondria and continuous membranes. The majority of cryoinjuries were observed commonly in preantral and antral follicles from vitrified/grafted ovaries and included nuclei shrinkage, damaged basement membrane of granulosa cells, irregular distribution of cytoplasmic organelles together with the accumulation of vacuoles. The ultrastructure of the preantral and antral follicles from ovarian tissue vitrified/grafted with DCV1 was well preserved and showed great similarity with the control group (Fig.2).

**Fig.3:**
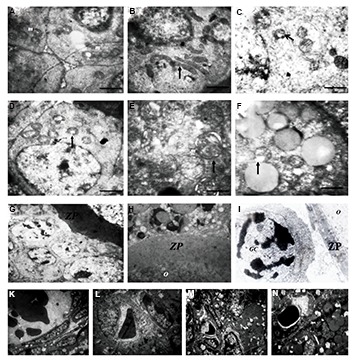
Ultrastructure of the mitochondria in antral follicle in murine ovarian tissue. **A.** Intact, **B.** Fresh grafted, **C.** Vitrified with DCV1/grafted, **D.** Vitrified with DCV2/grafted, **E.** Vitrified with DCV3/grafted, and **F.** Vitrified with DCV4/grafted. Ultrastructure of the zona pellucid (ZP) in the antral follicles: **G.** Vitrified with DCV1, **H.** Fresh grafted,** I.** Vitrified with DCV1/grafted. Ultrastructure of the blood vessels in murine ovarian tissue: **K.** Vitrified with DCV1/grafted, **L.** Vitrified with DCV2/grafted, **M.** Vitrified with DCV3/grafted, and **N.** Vitrified with DCV4/grafted (scale bar: 500 ىm). Numerous swollen mitochondria (m) and elongated mitochondria (black arrow) with a few cristae were observed in vitrified ovaries after transplantation. Damaged ZP was observed between oocyte (O) and granulosa cells (GC) inantral follicles. Vascular endothelial cells in the vessel lumen are detected frequently in vitrified ovarian tissue after transplantation.

The ultrastructure of the follicles was well-preserved in the control group (Fig.3A) and swollen mitochondria with a few cristae were found in antral follicles from fresh/ grafted ovaries (Fig.3B). Mitochondria showed the most signs of malformation and vacuolization in preantral and antral follicles from vitrified/grafted ovaries (Fig.3C-F). Theseirregularly shaped mitochondria were prominent in vitrified/grafted ovaries with DCV2, DCV3 and DCV4 (Fig.3D-F). Damaged zona pellucida was observed in some follicles from vitrified/grafted ovaries, in which wider empty spaces between the oocyte and the granulosa cells were identified (Fig.3G-I). Moreover, numerous blood vessels were detected in vitrified ovarian tissue after transplantation (Fig.3K), but newly formed blood vessels were frequently observed in ovaries vitrified/grafted with DCV1 (Fig.3L-N).

### Immunohistochemical analysis

**Fig.4:**
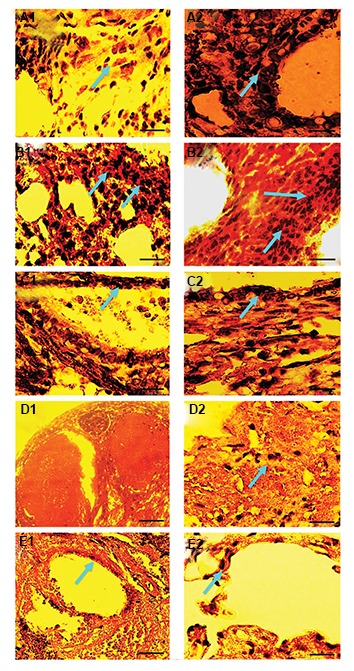
Immunohistochemical staining of new blood vessels in vitrified ovarian tissue after transplantation by the expression of ل-SMA and vWF. A1**.** α-SMA and **A2.** vWF expression in non-vitrified/grafted ovaries. **B1.** α-SMA and **B2.** vWF expression in vitrified with DCV1/grafted. **C1.** α-SMA and **C2.** vWF expression in DCV2-vitrified with /grafted. **D1.** α-SMA and **D2.** vWF expression in vitrified with DCV3/grafted. **E1.** α-SMA and **E2.** vWF expression in vitrified with DCV4/grafted ovarian tissue (scale bar: 10 μm). The expression of α-SMA and vWF was more prominent in cryopreserved ovaries with DCV1 following autotransplantation in comparison with the other groups. The positive cells are indicated by blue arrows.

Re-vascularization was detectable 7 days after autotransplantation of vitrified ovarian tissue, as indicated by the expression of α-SMA as a marker of smooth muscle cells or vWF as a marker of endothelial cells (Fig.4). Immunohistochemical analysis showed that the expression of vWF and α-SMA was more prominent in the ovarian tissue vitrified with DCV1 than in the vitrified ovarian tissue with DCV2, DCV3, and DCV4 after transplantation. This observation indicated that with regard to vascularization DCV1 was the suitable protocol for potent revascularization in vitrified ovarian tissue after transplantation.

### Real-time polymerase chain reaction analysis

**Fig.5:**
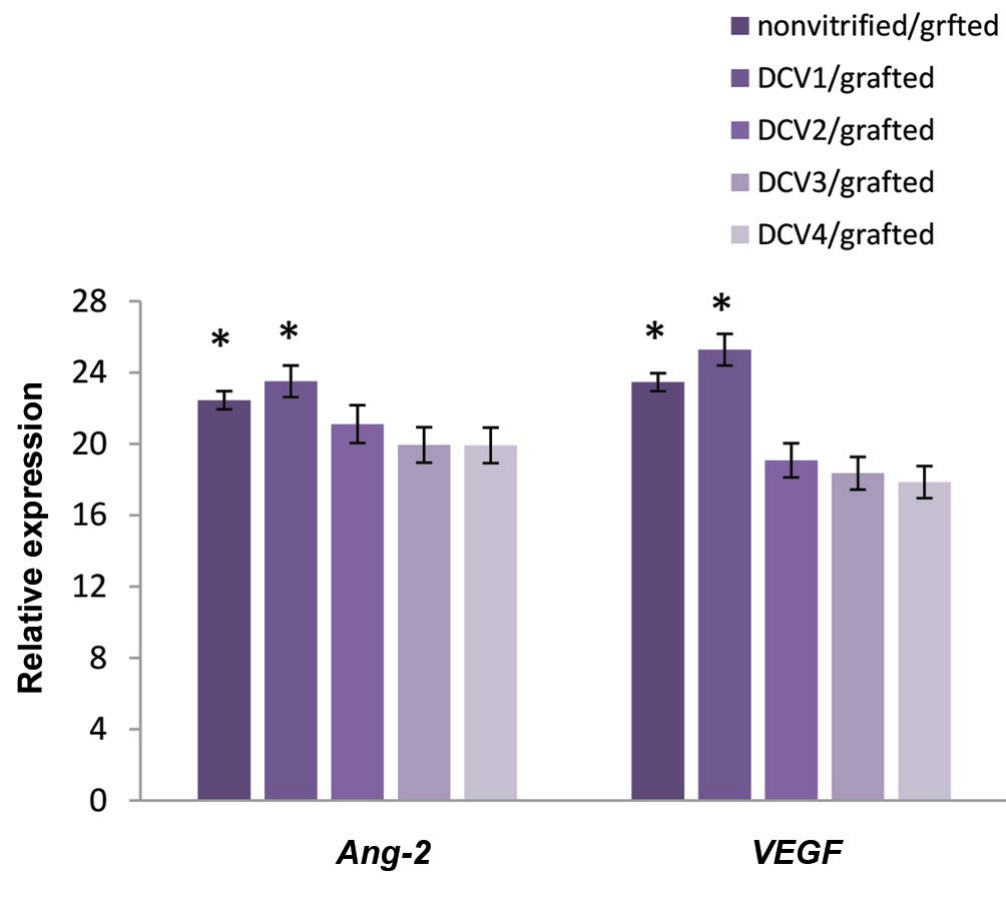
The expression levels of *VEGF* and *Ang-2* in vitrified ovarian tissue after transplantation by real time-polymerase chain reaction (PCR) analysis.*; Both angiogenic genes, *VEGF* and *Ang-2* were expressed at significantly higher levels in the ovarian tissue cryopreserved with DCV1 in comparison to the other groups after autotransplantation (P<0.01).

The expression levels of *VEGF* and *Ang-2* were detected in order to demonstrate the successful angiogenesis in vitrified/grafted ovarian tissue after transplantation (Fig.5). Real-time PCR analysis showed that *VEGF* and *Ang-2* genes were expressed in all vitrified/grafted ovaries following transplantation. The expression levels of both *VEGF* and *Ang-2* were increased significantly in ovarian tissue vitrified with DCV1 in comparison with the other vitrified ovaries post-transplantation (P<0.01). The levels of *VEGF* and *Ang-2* in DCV1 group did not differ from fresh ovaries.

## Discussion

Evaluation of angiogenesis has proven to be a useful tool for the assessment and comparison of the results of different CP agents and protocols on transplanted ovarian tissue. In addition, transplantation of ovaries provides a direct way to examine follicular viability and development during cryopreservation. In this study, it was shown that the histological features and ultrastructure of follicles were better preserved in ovarian tissue vitrified by EG and DMSO in a concentration of 10% after transplantation. Furthermore, high expression levels of VEGF and Ang- 2, which are crucial angiogenic factors, were observed in the cryopreserved ovarian tissue after subcutaneous ovary autotransplantation.

The current study illustrated that more histological degeneration was observed in oocyte and granulosa cells from ovaries vitrified/grafted in EG and DMSO mixture at a concentration of 15%. The evidence of degeneration included detachment of the innermost granulosa cells from the oocyte, oocyte shrinkage, and disarrangement of granulosa cells. The consistency that was observed between the ultrastructure and histological data confirmed that the organelles of granulosa cells and oocytes were well-preserved in the ovaries vitrified/grafted in DCV1. The greatest extent of follicular damage occurred during autotransplantation in cryopreserved ovarian tissue when a higher concentration of CP agent was used. These damages included vacuolization, lipid droplet distribution and swollen mitochondria with abnormal cristae. In this study, the grafted ovarian tissue following vitrification with DCV1 showed a remarkable microvessel density compared to the other vitrification protocols due to the reduction in cryoinjury.

Vitrification is a useful technique for cryopreservation of ovarian tissue (3-7). Nonetheless, CP toxicity is a main challenge in vitrification, but it can be limited by manipulating the nature and concentrations of the CP agents as well as the cooling rate (4, 6, 7, 10, 11). A fast cooling rate has been achieved in the DCV protocol, where ovarian tissue is plunged directly into liquid nitrogen. The current study suggests that the optimal vitrification concentration appears to play an important role in ovarian tissue cryopreservation. Therefore, a critical challenge in cryobiology is to obtain an optimal concentration of CP with low biological toxicity (25).

Our study in contrast with the Leonel et al. (26) results indicated that using the mixed cryopreservation technique by a stepwise addition of CP agents provides the best condition for cryopreservation of mouse ovarian tissue. These conflicting results may be explained by differences in protocols, CP solutions and concentrations, exposure times, or species. In the current study, follicular integrity at various stages of development was preserved in the cryopreserved ovarian tissue by using 10% EG and 10% DMSO through sufficient dehydration and penetration into ovarian tissue. However, vitrification procedures need to be optimized to minimize follicular failure after cryopreservation and grafting of ovarian tissue.

In this study, mitochondria were commonly damaged during the vitrification and transplantation of ovarian tissues. The mitochondrial organization is necessary for producing energy, Ca^2+^ signaling, regulating cell viability and cell function (27, 28). Several studies have indicated that the damaged mitochondria following vitrification may lead to in appropriate metabolic activity, reduced ATP levels, or disruption of the cytoskeleton proteins, and thereby result in free radical production (29).

Dare et al. (30) demonstrated that prolonged cryopreservation of the heart increased mitochondrial free radical production and ischemia-reperfusion damage during grafting. Therefore, it is suggested that ischemic and hypoxia conditions are the main causes of follicular loss after ovarian tissue transplantation, due to the production of free radicals (31, 32). It can be assumed that mitochondrial damage during vitrification and transplantation not only induces free radical production and apoptosis, but also causes ischemic conditions, and thereby leads to delayed revascularization.

In this study, it was found that the microvasculature distribution occurs mainly within the first week after transplantation based on observations of the ultrastructure and immunohistochemical examination. In particular, the markers of the vascular endothelial cells and pericyte cells were prominently found in ovarian tissue vitrified and grafted with EG and DMSO in concentrations of 10%, suggesting that the vascularization had returned to normal levels.

According to real-time PCR results, expression of *VEGF* and *Ang-2* was detected in the vitrifying and grafting ovarian tissues, which explains the promotion of angiogenesis in ovarian tissue post-transplantation. A significantly high level of *VEGF* expression was observed after transplantation in the ovarian tissue vitrified by 10% EG and 10% DMSO. Therefore, *VEGF* seems to be a critical index for predicting the success rate of vitrified/ grafted ovaries and may result in the improvement of follicular integrity preservation during transplantation. Furthermore, it was found that the expression of *VEGF* and *Ang-2* was diminished in cryopreserved ovarian tissue that had high concentrations of CP, suggesting that angiogenesis is adversely disrupted by vitrification and transplantation, and thus reduces ovarian function due to the initial ischemia.

It appears that VEGF, Ang-1 and Ang-2 as angiogenic factors are expressed in endothelial cells under normal or pathological conditions (33). The most recognized angiogenic factor expressed in ovarian tissue is VEGF (34). It was hypothesized that high expression levels of VEGF induce the formation of new blood vessels after ovarian tissue transplantation and that Ang-2 promotes neovascularization and stabilizes new blood vessels in the presence of VEGF (35). These findings suggest that there is a positive correlation between the expression of angiogenic factors and the endothelial cell markers in grafted ovaries. Therefore, high expression of VEGF and Ang-2 can predict the success of a cryopreservation protocol and agent on ovarian tissue during vitrification and grafting. In accordance with a study by Lee et al. (16), our current results have demonstrated that ovarian tissue subjected to a combination of vitrification and transplantation procedures may suffer from massive follicular damage during the initial days after ovarian transplantation. Grafting was much more detrimental to ovarian tissue than the vitrification procedure because of delayed tissue revascularization. Moreover, it was observed that large follicles including preantral and antral follicles were much more vulnerable to ischemic damage due to the high level of metabolite activity and delayed revascularization (36).

The main limitation in transplantation is ischemic reperfusion damage (37) and graft site plays a critical role in the revascularization of grafting tissue. Expressed angiogenic factors at a graft site induced the formation of new blood vessels in the grafted ovaries, and the improvement of angiogenesis could be an efficient strategy for evaluating a cryopreservation protocol (14). The results of Schubert et al. (14) suggested that the subcutaneous space is a convenient heterotopic transplantation site for the restoration of ovarian function and provision of follicular growth. In agreement with the results of Yang et al. (38), our data suggested that ovarian tissue autografted to a subcutaneous site could preserve follicular viability and ovarian function as a result of revascularization in the grafted site.

Although the subcutaneous area does not have a high blood supply, it was selected because it was easier to observe and follow up the situation of the grafted ovarian tissue and the transplantation surgery was easy to perform in experiments using murine models (14, 16). Revascularization is very important to minimizing ischemia damage,thereby facilitating angiogenesis and restoring ovarian function after transplantation in vitrified ovaries are crucial. Moreover, angiogenesis and revascularization in the early stage of transplantation are essential for follicular preservation and viability, allowing the follicular cells to provide oxygen and other vital factors (36). The current results are in agreement with previous studies, which illustrated that angiogenesis in vitrified ovaries initiates within 48 hours after transplantation and is elevated during 7 days after transplantation by an increase in expression of angiogenic factors (39).

It has been indicated that VEGF protects granulosa cells from undergoing apoptosis during ovarian cryopreservation and that it maintains the follicular pool (40). Interestingly, the current results suggest that VEGF and Ang-2 have a synergistic effect on the revascularization of cryopreserved ovarian tissue after grafting. Moreover, the expression of VEGF and Ang-2 is critical for the evaluation of optimal CP agents in the cryopreservation of ovarian tissue.

## Conclusion

The present study demonstrated that DCV is an effective protocol for cryopreservation of ovarian tissue. In addition, the solution of EG and DMSO in concentrations of 10% is the most efficacious CP agent for preserving follicular viability and development after transplantation of ovarian tissue, because it facilitates angiogenesis and improves revascularization capability through higher expression of angiogenic factors. However, further research is needed to optimize the vitrification processes to preserve follicular integrity in grafted ovaries.

## References

[1] Donnez J, Dolmans MM, Pellicer A, Diaz-Garcia C, Sanchez Serrano M, Schmidt KT (2013). Restoration of ovarian activity and pregnancy after transplantation of cryopreserved ovarian tissue: a review of 60 cases of reimplantation. Fertil Steril.

[2] Mathias FJ, DʹSouza F, Uppangala S, Salian SR, Kalthur G, Adiga SK (2014). Ovarian tissue vitrification is more efficient than slow freezing in protecting oocyte and granulosa cell DNA integrity. Syst Biol Reprod Med.

[3] Ramezani M, Salehnia M, Jafarabadi M (2018). Vitrification and in vitro culture had no adverse effect on the follicular development and gene expression of stimulated human ovarian tissue. J Obstet Gynaecol Res.

[4] Li YB, Zhou CQ, Yang GF, Wang Q, Dong Y, Morrow T (2007). Modified vitrification method for cryopreservation of human ovarian tissues. Chin Med J (Engl).

[5] Zhang JM, Li LX, Liu XL, Yang YX, Wan XP (2009). Sucrose affecting successful transplantation of vitrified-thawed mouse ovarian tissues. J Assist Reprod Genet.

[6] Nateghi R, Alizadeh A, JafariAhangari Y, Fathi R, Akhlaghi A (2017). Ethylene glycol and dimethyl sulfoxide combination reduces cryoinjuries and apoptotic gene expression in vitrified laying hen ovary. Biopreserv Biobank.

[7] Youm HW, Lee JR, Lee J, Jee BC, Suh CS, Kim SH (2014). Optimal vitrification protocol for mouse ovarian tissue cryopreservation: effect of cryoprotective agents and in vitro culture on vitrified-warmed ovarian tissue survival. Hum Reprod.

[8] Lima GL, Luz VB, Lunardi FO, Souza ALP, Peixoto GCX, Rodrigues APR (2019). Effect of cryoprotectant type and concentration on the vitrification of collared peccary (Pecaritajacu) ovarian tissue. Anim Reprod Sci.

[9] Ladanyi C, Mor A, Christianson MS, Dhillon N, Segars JH (2017). Recent advances in the field of ovarian tissue cryopreservation and opportunities for research. J Assist Reprod Genet.

[10] Chen SU, Chien CL, Wu MY, Chen TH, Lai SM, Lin CW (2006). Novel direct cover vitrification for cryopreservation of ovarian tissues increases follicle viability and pregnancy capability in mice. Hum Reprod.

[11] Zhou XH, Wu YJ, Shi J, Xia YX, Zheng SS (2010). Cryopreservation of human ovarian tissue: comparison of novel direct cover vitrification and conventional vitrification. Cryobiology.

[12] Ghavami M, Mohammadnejad D, Beheshti R, Solmani-rad J, Abedelahi A (2015). Ultrastructural and morphalogical changes of mouse ovarian tissues following direct cover vitrification with different cryoprotectants. J Reprod Infertil.

[13] Tayefi-Nasrabadi H, Gavami M, Akbarzadeh A, Beheshti R, Mohammadnejad D, Abedelahi A (2015). Preservation of mouse ovarian tissue follicle morphology and ultra-structure after vitrifying in biotechnological protocols. J Ovarian Res.

[14] Schubert B, Canis M, Darcha C, Artonne C, Smitz J, Grizard G (2008). Follicular growth and estradiol follow-up after subcutaneous xenografting of fresh and cryopreserved human ovarian tissue. Fertil Steril.

[15] Gao J, Huang Y, Li M, Zhao H, Zhao Y, Li R (2015). Effect of local basic fibroblast growth factor and vascular endothelial growth factor on subcutaneously allotransplanted ovarian tissue in ovariectomized mice. PLoS One.

[16] Lee J, Kong HS, Kim EJ, Youm HW, Lee JR, Suh CS (2016). Ovarian injury during cryopreservation and transplantation in mice: a comparative study between cryoinjury and ischemic injury. Hum Reprod.

[17] Cheraghi O, Dehghan G, Mahdavi M, Rahbarghazi R, Rezabakhsh A, Charoudeh HN (2016). Potent anti-angiogenic and cytotoxic effect of conferone on human colorectal adenocarcinoma HT-29 cells. Phytomedicine.

[18] Siavashi V, Nassiri SM, Rahbarghazi R, Vafaei R, Sariri R (2016). ECM‐ Dependence of endothelial progenitor cell features. J Cell Biochem.

[19] Mattioli M, Barboni B, Turriani M, Galeati G, Zannoni A, Castellani G (2001). Follicle activation involves vascular endothelial growth factor production and increased blood vessel extension. Biol Reprod.

[20] Araújo VR, Duarte AB, Bruno JB, Pinho Lopes CA, de Figueiredo JR (2013). Importance of vascular endothelial growth factor (VEGF) in ovarian physiology of mammals. Zygote.

[21] Asadi E, Najafi A, Moeini A, Pirjani R, Hassanzadeh G, Mikaeili S (2017). Ovarian tissue culture in the presence of VEGF and fetuin stimulates follicle growth and steroidogenesis. J Endocrinol.

[22] Lin Z, Liu Y, Sun Y, He X (2011). Expression of Ets-1, Ang-2 and maspin in ovarian cancer and their role in tumor angiogenesis. J Exp Clin Cancer Res.

[23] Zanetta L, Marcus SG, Vasile J, Dobryansky M, Cohen H, Eng K (2000). Expression of von Willebrand Factor, an endothelial cell marker, is up-regulated by angiogenesis factors: a potential method for objective assessment of tumor angiogenesis. Int J Cancer.

[24] Candy CJ, Wood MJ, Whittingham DG (1997). Effect of cryoprotectants on the survival of follicles in frozen mouse ovaries. J Reprod Fertil.

[25] Courbiere B, Odagescu V, Baudot A, Massardier J, Mazoyer C, Salle B (2006). Cryopreservation of the ovary by vitrification as an alternative to slow-cooling protocols. Fertil Steril.

[26] Leonel ECR, Vilela JMV, Carrilho DJ, Lucci CM (2018). Cat ovarian follicle ultrastructure after cryopreservation with ethylene glycol and dimethyl sulfoxide. Cryobiology.

[27] Liu L, Hammar K, Smith P, Inoue S, Keefe DL (2001). Mitochondrial modulation of calcium signaling at the initiation of development. Cell Calcium.

[28] Baril G, Traldi AL, Cognié Y, Lebeouf B, Beckers JF, Mermillod P (2001). Successful direct transfer of vitrified sheep embryos. Theriogenology.

[29] Zampolla T, Spikings E, Srirangarajah S, Rawson DM, Zhang T (2011). Impact of cryoprotectants and cryopreservation on metabolic activity and cytoskeleton proteins of zebrafish (Danio rerio) ovarian fragments. Cryo Letters.

[30] Dare AJ, Logan A, Prime TA, Rogatti S, Goddard M, Bolton EM (2015). The mitochondria-targeted anti-oxidant MitoQ decreases ischemia-reperfusion injury in a murine syngeneic heart transplant model. J Heart Lung Transplant.

[31] Abedelahi A, Salehnia M, Allameh A, Davoodi D (2010). Sodium selenite improves the in vitro follicular development by reducing the reactive oxygen species level and increasing the total antioxidant capacity and glutathione peroxide activity. Hum Reprod.

[32] Jassem W, Fuggle SV, Rela M, Koo DD, Heaton ND (2002). The role of mitochondria in ischemia/reperfusion injury. Transplantation.

[33] Rahbarghazi R, Nassiri SM, Ahmadi SH, Mohammadi E, Rabbani S, Araghi A (2014). Dynamic induction of pro-angiogenic milieu after transplantation of marrow-derived mesenchymal stem cells in experimental myocardial infarction. Int J cardiol.

[34] Hazzard TM, Molskness TA, Chaffin CL, Stouffer RL (1999). Vascular endothelial growth factor (VEGF) and angiopoietin regulation by gonadotrophin and steroids in macaque granulosa cells during the peri-ovulatory interval. Molecular Hum Reprod.

[35] Asahara T, Chen D, Takahashi T, Fujikawa K, Kearney M, Magner M (1998). Tie2 receptor ligands, angiopoietin-1 and angiopoietin-2, modulate VEGF-induced postnatal neovascularization. Circ Res.

[36] Liu J, Van der Elst J, Van den Broecke R, Dhont M (2002). Early massive follicle loss and apoptosis in heterotopically grafted newborn mouse ovaries. Hum Reprod.

[37] Bedaiwy MA, Jeremias E, Gurunluoglu R, Hussein MR, Siemianow M, Biscotti C (2003). Restoration of ovarian function after autotransplantation of intact frozen-thawed sheep ovaries with microvascular anastomosis. Fertil Steril.

[38] Yang H, Lee HH, Lee HC, Ko DS, Kim SS (2008). Assessment of vascular endothelial growth factor expression and apoptosis in the ovarian graft: can exogenous gonadotropin promote angiogenesis after ovarian transplantation?. Fertil Steril.

[39] Wu D, Lei Y, Tong Y, Tang F, Qian Y, Zhou Y (2010). Angiogenesis of the frozen‐thawed human fetal ovarian tissue at the early stage after xenotransplantation and the positive effect of salviae miltiorrhizae. Anat Rec (Hoboken).

[40] Roberts AE, Arbogast LK, Friedman CI, Cohn DE, Kaumaya PT, Danforth DR (2007). Neutralization of endogenous vascular endothelial growth factor depletes primordial follicles in the mouse ovary. Biol Reprod.

